# Tobacco Smoke, Indoor Air Pollution and Tuberculosis: A Systematic Review and Meta-Analysis

**DOI:** 10.1371/journal.pmed.0040020

**Published:** 2007-01-16

**Authors:** Hsien-Ho Lin, Majid Ezzati, Megan Murray

**Affiliations:** 1 Department of Epidemiology, Harvard School of Public Health, Boston, Massachusetts, United States of America; 2 Department of Population and International Health and Department of Environmental Health, Harvard School of Public Health, Boston, Massachusetts, United States of America; 3 Division of Social Medicine and Health Inequalities, Brigham and Women's Hospital, Boston, Massachusetts, United States of America; 4 Infectious Disease Unit, Massachusetts General Hospital, Boston, Massachusetts, United States of America; Center for Tobacco Control Research and Education, United States of America

## Abstract

**Background:**

Tobacco smoking, passive smoking, and indoor air pollution from biomass fuels have been implicated as risk factors for tuberculosis (TB) infection, disease, and death. Tobacco smoking and indoor air pollution are persistent or growing exposures in regions where TB poses a major health risk. We undertook a systematic review and meta-analysis to quantitatively assess the association between these exposures and the risk of infection, disease, and death from TB.

**Methods and Findings:**

We conducted a systematic review and meta-analysis of observational studies reporting effect estimates and 95% confidence intervals on how tobacco smoking, passive smoke exposure, and indoor air pollution are associated with TB. We identified 33 papers on tobacco smoking and TB, five papers on passive smoking and TB, and five on indoor air pollution and TB. We found substantial evidence that tobacco smoking is positively associated with TB, regardless of the specific TB outcomes. Compared with people who do not smoke, smokers have an increased risk of having a positive tuberculin skin test, of having active TB, and of dying from TB. Although we also found evidence that passive smoking and indoor air pollution increased the risk of TB disease, these associations are less strongly supported by the available evidence.

**Conclusions:**

There is consistent evidence that tobacco smoking is associated with an increased risk of TB. The finding that passive smoking and biomass fuel combustion also increase TB risk should be substantiated with larger studies in future. TB control programs might benefit from a focus on interventions aimed at reducing tobacco and indoor air pollution exposures, especially among those at high risk for exposure to TB.

## Abstract 2

Evidence from a number of studies suggest that tobacco smoking, environmental tobacco smoke, and indoor air pollution from biomass fuels is associated with an increased risk of tuberculosis.

## Editors' Summary

**Background.:**

Tobacco smoking has been identified by the World Health Organization as one of the leading causes of death worldwide. Smokers are at higher risk than nonsmokers for a very wide variety of illnesses, many of which are life-threatening. Inhaling tobacco smoke, whether this is active (when an individual smokes) or passive (when an individual is exposed to cigarette smoke in their environment) has also been associated with tuberculosis (TB). Many people infected with the TB bacterium never develop disease, but it is thought that people infected with TB who also smoke are far more likely to develop the symptoms of disease, and to have worse outcomes when they do.

**Why Was This Study Done?:**

The researchers were specifically interested in the link between smoking and TB. They wanted to try to work out the overall increase in risk for getting TB in people who smoke, as compared with people who do not smoke. In this study, the researchers wanted to separately study the risks for different types of exposure to smoke, so, for example, what the risks were for people who actively smoke as distinct from people who are exposed to smoke from others. The researchers also wanted to calculate the association between TB and exposure to indoor pollution from burning fuels such as wood and charcoal.

**What Did the Researchers Do and Find?:**

In carrying out this study, the researchers wanted to base their conclusions on all the relevant information that was already available worldwide. Therefore they carried out a systematic review. A systematic review involves setting out the research question that is being asked and then developing a search strategy to find all the meaningful evidence relating to the particular question under study. For this systematic review, the researchers wanted to find all published research in the biomedical literature that looked at human participants and dealt with the association between active smoking, passive smoking, indoor air pollution and TB. Studies were included if they were published in English, Russian, or Chinese, and included enough data for the researchers to calculate a number for the increase in TB risk. The researchers initially found 1,397 research studies but then narrowed that down to 38 that fit their criteria. Then specific pieces of data were extracted from each of those studies and in some cases the researchers combined data to produce overall calculations for the increase in TB risk. Separate assessments were done for different aspects of “TB risk,” namely, TB infection, TB disease, and mortality from TB. The data showed an approximately 2-fold increase in risk of TB infection among smokers as compared with nonsmokers. The researchers found that all studies evaluating the link between smoking and TB disease or TB mortality showed an association, but they did not combine these data together because of wide potential differences between the studies. Finally, all studies looking at passive smoking found an association with TB, as did some of those examining the link with indoor air pollution.

**What Do These Findings Mean?:**

The findings here show that smoking is associated with an increased risk of TB infection, disease, and deaths from TB. The researchers found much more data on the risks for active smoking than on passive smoking or indoor air pollution. Tobacco smoking is increasing in many countries where TB is already a problem. These results therefore suggest that it is important for health policy makers to further develop strategies for controlling tobacco use in order to reduce the impact of TB worldwide.

**Additional Information.:**

Please access these Web sites via the online version of this summary at http://dx.doi.org/10.1371/journal.pmed.0040020

- The World Health Organization (WHO)'s Tobacco Free Initiative provides resources on research and policy related to tobacco control, its network of initiatives, and other relevant information
- WHO also has a tuberculosis minisite
- The US National Library of Medicine's MedLinePlus provides a set of links and resources about smoking, including news, overviews, recent research, statistics, and others
- The Health Consequences of Smoking: A Report of the Surgeon General provides information on the health consequences of smoking
- Tobacco Country Profiles provides information on smoking in different countries

## Introduction

Tuberculosis (TB) causes an estimated 2 million deaths per year, the majority of which occur in the developing world. Many studies conducted over the past 60 years have found an association between tobacco smoking and TB, as manifested by a positive tuberculin skin test (TST) or as active disease and its sequelae. A smaller number have found that indoor air pollution from biomass fuels (IAP) and passive smoking are also risk factors for TB and its sequelae. Tobacco smoking has increased substantially in developing countries over the past three decades, with an estimated 930 million of the world's 1.1 billion smokers currently living in the low-income and middle-income countries [1,2]. Approximately half of the world's population uses coal and biomass, in the form of wood, animal dung, crop residues, and charcoal as cooking and heating fuels especially in Africa and Asia. Given the persistent or growing exposure to both smoking and IAP in regions where TB poses a major health risk, it is essential to delineate the role of these environmental factors in the etiology and epidemiology of TB. Previous reviews have addressed qualitatively the epidemiologic and biologic link between tobacco smoke and TB, but have not systematically reviewed the epidemiologic data on this association [3,4]. We therefore undertook to quantitatively assess the association between smoking, passive smoking, and IAP, and the risk of infection, disease, and death from TB. We have considered smoking, passive smoking, and IAP together because these sources result in exposure to common set of respirable pollutants, and because their effects are currently or increasingly found in the developing countries.

## Methods

### Data Source

We searched the PubMed via the NCBI Entrez system (1950 to February 1, 2006) (http://www.ncbi.nlm.nih.gov/entrez/query.fcgi) and the EMBASE via Ovid (1988 to 2003) (http://www.ovid.com) for studies of the association between smoking, passive smoking, and indoor air pollution and TB infection, disease, and mortality. We also searched bibliographies of identified reports for additional references. Our search strategy is described in Box 1.

### Study Selection

We limited our search to studies published in English, Russian, and Chinese. Studies were included if they involved human participants with TB or at risk from TB. We included studies if a quantitative effect estimate of the association between ever, former, or current tobacco smoking, passive smoking, or IAP, and TST positivity, clinical TB disease, or TB mortality was presented or could be estimated from the data provided in the paper or through contact with the authors. Studies were included in the review if they were full-length peer-reviewed reports of cohort studies, case-control studies, or cross-sectional studies, if they controlled for possible confounding by age or age group, and if they screened for the presence of TB among exposed and unexposed study participants in the same way. For analyses of the effect of passive smoking on TB outcomes, we excluded studies if they did not restrict the population under study to nonsmokers. If multiple published reports from the same study participants were available, we included only the one with the most detailed information for both outcome and exposure.

**Box 1.**

Search Strategy and Terms Used to Identify Studies on Smoking and TB

**MeSH term search**

1. “tuberculosis”

2. “smoking”

3. “air pollution, indoor”

4. “biomass”

5. “fuel oils”

6. “(1) AND (2)” OR “(1) AND (3)” OR “(1) AND (4)” OR “(1) AND (5)”

**Direct keyword search:**

7. “tuberculosis”

8. “smoking”

9. “indoor air pollution”

10. “cooking fuel”

11. “biomass”

12. “(7) AND (8)” OR “(7) AND (9)” OR “(7) AND (10)” OR “(7) AND (11)”

13. (6) OR (12)

### Data Extraction and Quality Assessment

For every eligible study, we collected detailed information on year and country of study, study design, study population, sample size, choice of controls, definition and measurement of tobacco smoking or IAP, type of TB outcome, confounders adjusted for, effect sizes and 95% confidence intervals (CIs), and dose-response relationships. Since TB disease and death are relatively rare events, even in high-incidence areas, we assumed that odds ratios (ORs), risk ratios, and rate ratios all provided an equivalent estimate of risk and therefore reported them as ORs [5]. Although latent TB infection is not a rare event, each of the studies of latent TB infection estimated ORs and we therefore reported ORs for this outcome as well. Data were extracted independently by two of the investigators (HL and MM), and differences were resolved by discussion with a third (ME).

### Data Synthesis

We performed separate analyses for each exposure-outcome association that had been studied. Within each subanalysis we further stratified on different study designs. When more than one study used a specific study design, we assessed heterogeneity using the *I* ^2^ statistic described by Higgins et al. [6]. Because of the significant heterogeneity and different study designs within subgroups, we did not compute pooled effect measures [7]. Instead, we graphically presented each of the weighted point estimates and 95% CIs of effect estimates for individual studies within subanalyses. For the subanalysis in which we found no significant heterogeneity, effect estimates were given a weight equal to the inverse variance of the study (fixed effects model). For those subanalyses in which we noted significant heterogeneity, we used a random effects model to assign the weight of each study according to the method described by DerSimonian and Laird [8]. In order to assess the effect of study quality on the reported effect estimates, we conducted sensitivity analyses in which we compared pooled effect estimates for subgroups stratified on quality-associated study characteristics including study design (cohort, case-control or cross-sectional), type of control selection (population based or other), adjustment for important potential confounder (alcohol and socioeconomic status), and outcome classification (microbiological or other). We considered studies to be of higher quality if they (1) were cohort studies, (2) were case-control studies using population-based controls, (3) adjusted for important confounders, (4) classified the outcome on the basis of microbiological findings, and (5) restricted the outcome to pulmonary TB. As above, pooled estimates were calculated using a fixed effects model if there was no significant heterogeneity and a random effects models for those subanalyses in which we found heterogeneity.

We tested for possible publication bias using Begg's and Egger's tests and by visual inspection for asymmetry of a plot of the natural logarithms of the effect estimates against their standard errors according to method described by Begg [9,10]. Several large studies on smoking and TB mortality had highly variable results and thus fell outside the lines of the funnel plot. Therefore, we conducted a sensitivity analysis in which we repeated the funnel plot excluding all of the mortality studies. All statistical procedures were carried out in Intercooled Stata Version 8.2 (Stata, http://www.stata.com).

## Results

**Figure 1:**
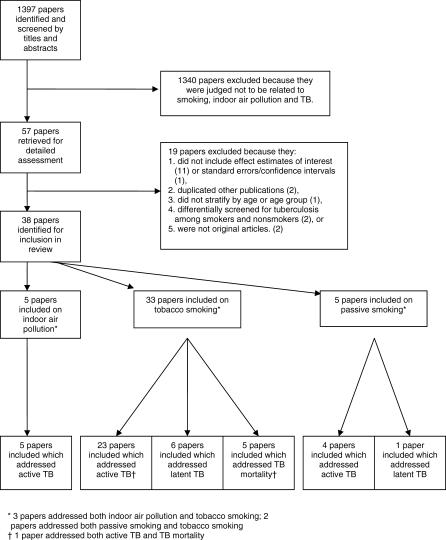
Flow Diagram of Study Steps and Exclusions

We identified and screened 1,397 papers by titles and abstracts. We excluded 1,340 papers because they were judged not to be related to smoking, IAP, and TB. The remaining 57 articles were obtained for detailed review; 19 of these were excluded because the same studies were published in different journals [11,12], the effect sizes and CIs of interest were not reported or could not be estimated [13–24], there were severe flaws in study design [25–27], or the article was not original [28,29]. Thirty-eight papers were included in the final analysis. Figure 1 delineates the exclusion process and Table 1 summarizes the studies that were included in the final analysis.

**Table 1:**
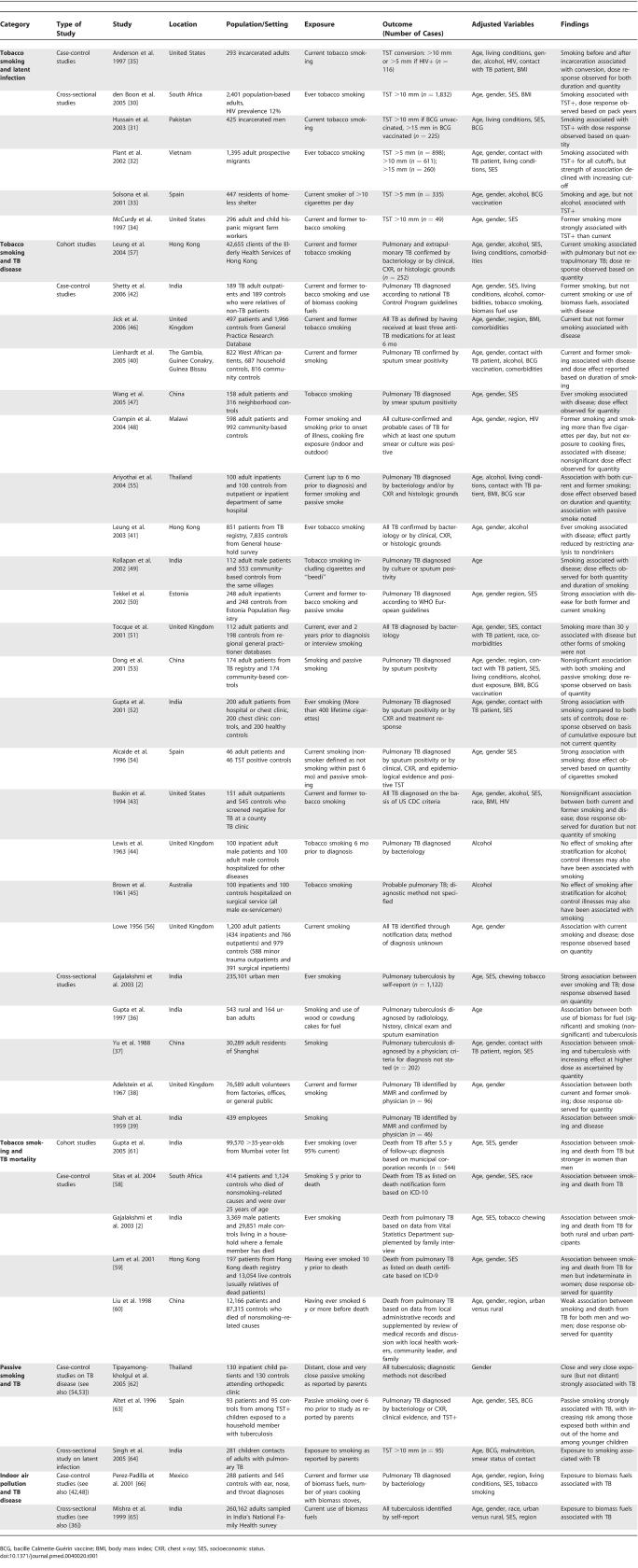
Study Characteristics

### Tobacco Smoking and Latent TB Infection

**Figure 2:**
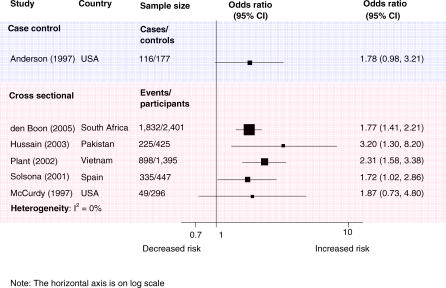
Risk of Latent TB Infection for Smoking Compared with Nonsmoking

Figure 2 shows the risk of latent TB among smokers compared with nonsmokers in six studies [30–35] on tobacco smoking and latent infection. The studies were conducted in five countries: the US, Spain, South Africa, Pakistan, and Vietnam. Although the timing of smoking (current, former, and ever) in relation to the study varied, we did not differentiate between these reported exposures, because the actual time of TB infection was unknown. There was only one case-control study; for the five cross-sectional studies that were included, we found minimal heterogeneity (*I* ^2^ = 0%). We also stratified studies that used different cutoffs for the TST; among those analyses that used induration size of 5 mm as the cutoff for a positive test [32,33], the pooled OR for latent TB was 2.08 (95% CI, 1.53–2.83), while among those that used a 10 mm cutoff [30,31,34,35], the pooled OR was 1.83 (95% CI, 1.49–2.23). When we stratified on other quality-associated study characteristics, we found that ORs for TB infection were lower among studies that adjusted for alcohol (Table 2), but that a positive effect of smoking on latent TB remained.

**Table 2:**
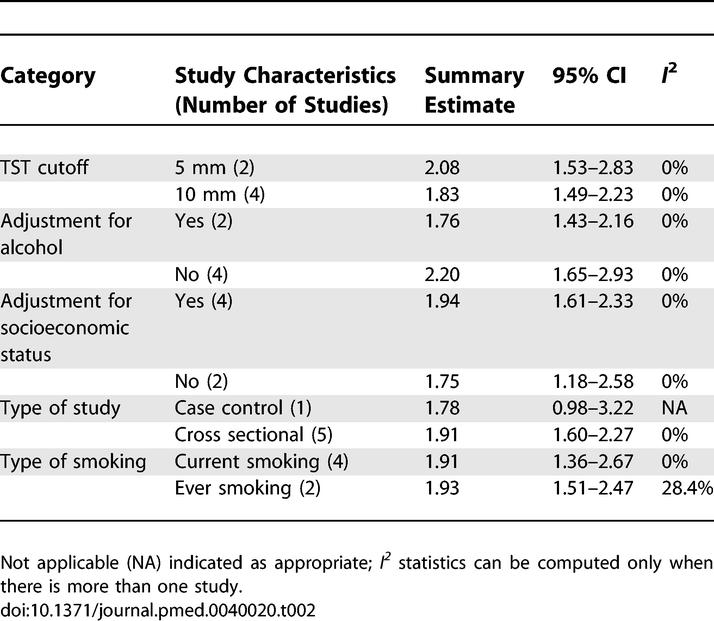
Quality Assessment and Subgroup Analysis: Tobacco Smoking and Latent TB Infection

### Tobacco Smoking and Clinical TB Disease

**Figure 3:**
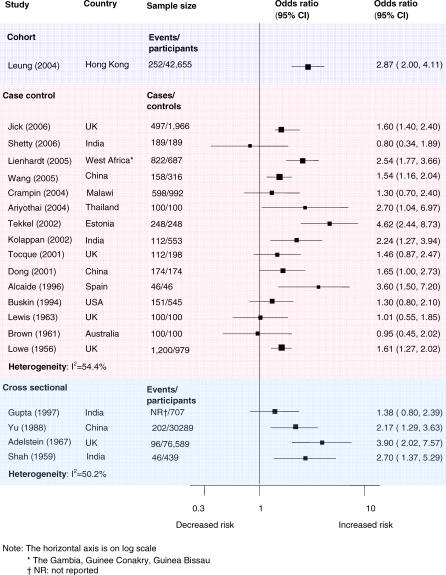
Risk of Clinical TB Disease for Current Smoking Compared with Nonsmoking

**Figure 4:**
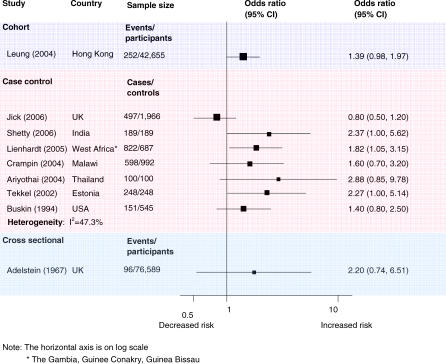
Risk of Clinical TB Disease for Former Smoking Compared with Nonsmoking

**Figure 5:**
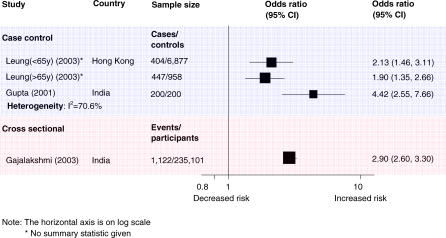
Risk of Clinical TB Disease for Ever Smoking Compared with Nonsmoking

The 23 studies that we identified on the association between tobacco smoking and clinical TB disease were conducted in 12 countries: China/Hong Kong, India, The Gambia, Guinee Conakry, Guinea Bissau, US, UK, Australia, Malawi, Estonia, Spain, and Thailand [2,36–57]. Figures 3–5 shows the risk of clinical TB among current, former, and ever smokers, respectively, compared to nonsmokers for the individual studies. Given the significant heterogeneity among each of these effect estimates, we do not report pooled estimates within each of these three categories; rather, we stratified on important study characteristics within each category for the purpose of sensitivity analysis (Table 3). These analyses show that there was a significantly increased risk of clinical TB among smokers regardless of outcome definition (pulmonary TB versus any TB), adjustment for alcohol intake or socioeconomic status, type of study, or choice of controls. Although stratification by these study-specific variables did not fully explain the variability between studies, heterogeneity was partially accounted for by outcome (pulmonary versus any TB) and by adjustment for alcohol intake. As might be predicted on the basis of biological plausibility, we found a higher risk of clinical TB among smokers when we restricted the analyses to studies that included only cases of pulmonary disease. However, the differences between the effect estimates for pulmonary TB and those for any TB were not statistically significant.

**Table 3:**
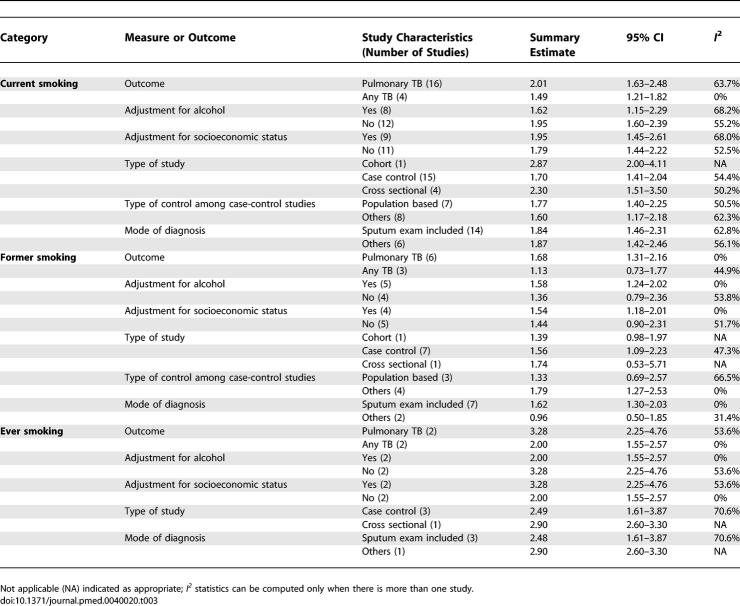
Quality Assessment and Subgroup Analysis: Tobacco Smoking and TB Disease

### Tobacco Smoking and TB Mortality

**Figure 6:**
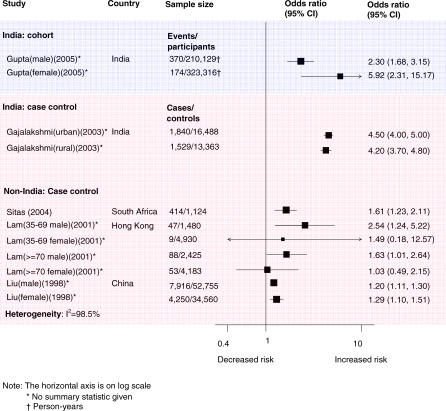
Risk of Mortality Due to TB for Smoking Compared with Nonsmoking

We identified five studies on tobacco smoking and TB mortality in adults [2,58–61], conducted in India, South Africa, and China/Hong Kong. Although all of the studies found a positive association between smoking and TB mortality (Figure 6), there was substantial heterogeneity (*I* ^2^ = 98.5% among case-control studies) and a five-fold difference between the most extreme effect estimates. We therefore do not report a pooled estimate for this analysis. A dose-response relation was noted in the two [59,60] studies that stratified on dose. When we conducted a sensitivity analysis excluding the study conducted in India where TB may have been differentially overdetected among smokers [2,61], heterogeneity was markedly reduced (*I* ^2^ = 38.6%). Other sensitivity analyses are demonstrated in Table 4.

**Table 4:**
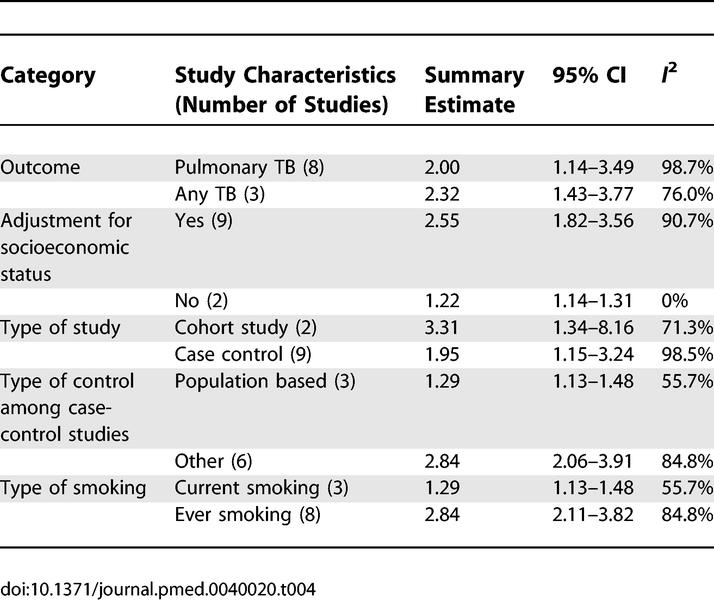
Quality Assessment and Subgroup Analysis: Tobacco Smoking and TB Mortality

### Passive Smoking and TB

**Figure 7:**
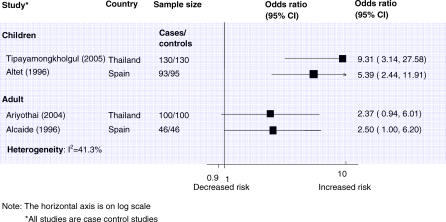
Risk of Clinical TB Disease for Passive Smoking Exposure Compared with Nonexposure

We identified five studies on passive smoking and TB, of which four were case-control studies assessing the risk of clinical TB [50,53–55,62,63] and one a cross-sectional study on the risk of latent infection [64]. Two studies did not exclude active smokers while assessing passive smoking and were, therefore, not included in the analysis of passive smoking and TB [50,53]. Figure 7 shows the individual effect measures for the studies on active disease; each found a positive association between passive smoking and TB. The heterogeneity among the studies was largely explained by the age of the participants; the risk of TB among children exposed to passive smoking was significantly higher than it was among adults (*p* = 0.002), and there was no remaining heterogeneity within the subgroups stratified by age. The single study examining the risk of latent TB infection among those exposed to passive smoking reported an OR of 2.68 (95% CI, 1.52–4.71) [64]. Sensitivity analyses for these estimates are given in Table 5.

**Table 5:**
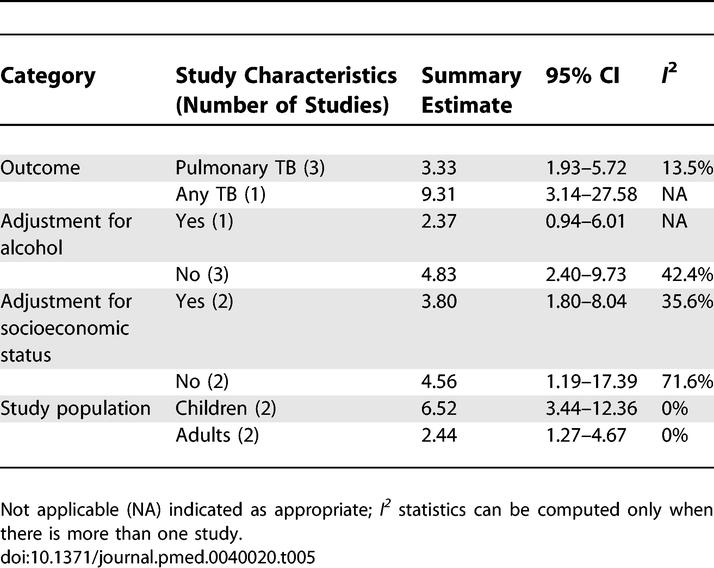
Quality Assessment and Subgroup Analysis: Passive Smoking and TB Disease

A dose response was found in both of the two studies that stratified on exposure intensity; one found that TB risk increased with the number of cigarettes smoked by the family per day [63], and the other found that close and very close contact with smoking household members was strongly associated with TB (adjusted OR 9.31 [95% CI, 3.14–27.58]), while distant contact was not (adjusted OR 0.54 [95% CI, 0.25–1.16]) [62].

### IAP and Clinical TB Disease

**Figure 8:**
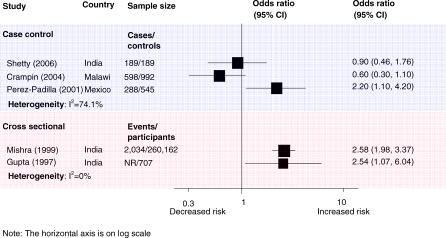
Risk of Clinical TB Disease for Indoor Air Pollution Exposure Compared with Nonexposure

Only five studies of IAP and TB were identified (Figure 8) [36,42,48,65,66]. Of these, only two studies adjusted for tobacco smoking [42,66] while three others did not [36,48,65]. In each of the studies, IAP was assessed by questionnaire on cooking and heating with biomass fuels (wood or dung). Although three of the five studies reported a positive association between biomass use and TB disease, there was significant heterogeneity among the studies (*I* ^2^ = 74.1% in case-control studies) (Figure 8). We noted that in one study, houses were reportedly well ventilated and therefore the impact of IAP might have been attenuated [48]. The sensitivity analyses are presented in Table 6.

**Table 6:**
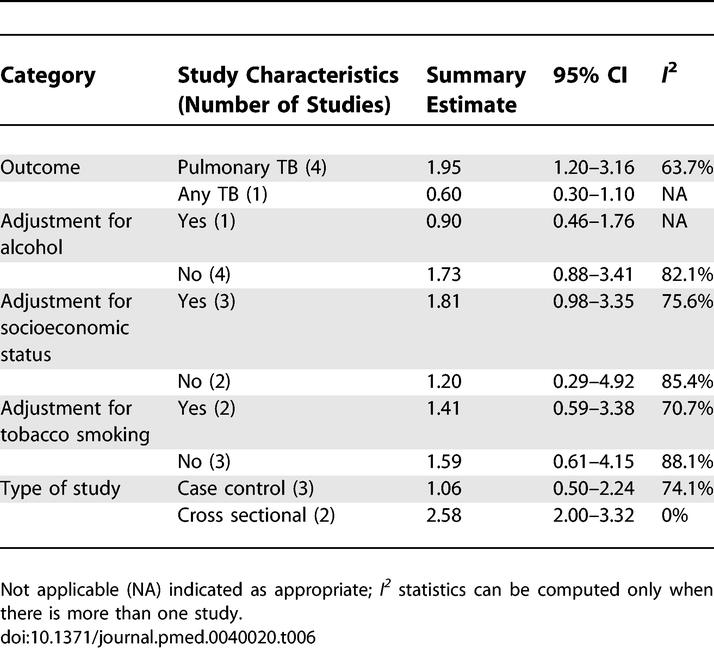
Quality Assessment and Subgroup Analysis: Indoor Air Pollution and TB Disease

### Publication Bias

**Figure 9:**
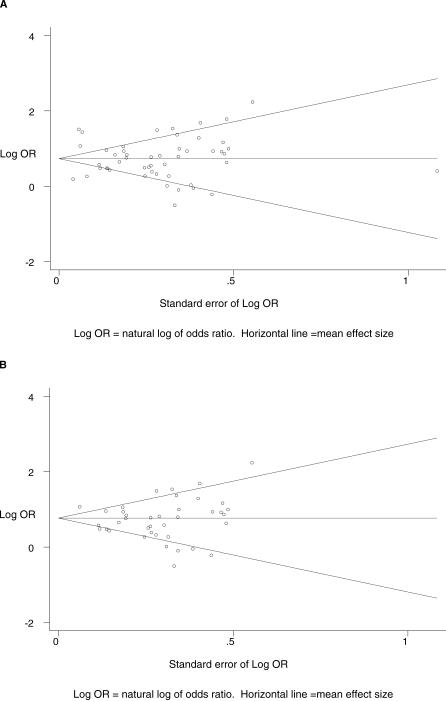
Begg's Funnel Plots with Pseudo 95% Confidence Limits (A) Funnel plot for all studies. (B) Funnel plot for all studies excluding mortality studies.

When we plotted the natural logarithms of the effect estimates against their standard errors using the methods described by Begg (Figure 9A) [9], we detected some slight asymmetry of effect estimates among small studies. We also noted that several large studies fell outside the projected lines of the funnel plot, indicating substantial variability among studies with small standard errors. When we repeated this analysis excluding the five mortality studies, we found that the studies with small standard errors clustered within the funnel plot (Figure 9B). We found no evidence for substantial publication bias by either the Begg's test (*p* = 0.256) or the Egger's test (*p* = 0.977).

## Discussion

This analysis shows that exposure to tobacco smoke is consistently associated with TB, regardless of the specific types of exposures and specific TB outcomes. Compared with people who do not smoke, smokers have an increased risk of a positive TST, of having active TB, and of dying from TB. Although there were fewer studies for passive smoking and IAP from biomass fuels, those exposed to these sources were found to have higher risks of active TB than those who are not exposed. An important finding of this study is the suggestion that the risk of TB among those exposed to passive smoking is especially high in children who are not normally at high risk for active disease. These findings support the hypothesis that exposure to respirable pollutants from combustion of tobacco and biomass fuels increases the risk of both TB infection and TB disease.

In addition to the positive association demonstrated here, multiples lines of evidence support a causal relationship between combustion smoke and TB. A dose–response relationship has been demonstrated in most of the studies that have stratified on dose; in this meta-analysis, we found that the risk of TB increases with both daily dose of cigarettes and duration of smoking. There is also accumulating evidence for the biological plausibility of this association. Chronic exposure to tobacco as well as to a number of environmental pollutants impairs the normal clearance of secretions on the tracheobronchial mucosal surface and may thus allow the causative organism, *Mycobacterium tuberculosis,* to escape the first level of host defenses, which prevent bacilli from reaching the alveoli [67]. Smoke also impairs the function of pulmonary alveolar macrophages (AMs), which are not only the cellular target of M. tuberculosis infection but also constitute an important early defense mechanism against the bacteria; AMs isolated from the lungs of smokers have reduced phagocytic ability and a lower level of secreted proinflammatory cytokines than do those from nonsmokers [68]. Recent work has suggested a novel mechanism for this effect; nicotine is hypothesized to act directly on nicotinic acetylcholine receptors on macrophages to decrease intracellular tumor necrosis factor-α production and thus impair intracellular killing of M. tuberculosis [69]. Wood smoke exposure in rabbits has also been shown to negatively affect antibacterial properties of AMs, such adherence to surfaces, ability to phagocytize bacteria, and intracellular bactericidal processes [70]. Boelaert and colleagues [71] have also proposed an alternative explanation for the impaired ability of macrophages from smokers to contain M. tuberculosis infection. These investigators noted that AMs from smokers have an markedly elevated iron content and that macrophage iron overload impairs defense against intracellular microorganisms through reduced production of both tumor necrosis factor-α and nitric oxide.

The available data support a causal link between smoke exposure and either an increased chance of acquiring TB or progression of TB to clinical disease. Our study shows that the risk of latent TB among smokers is quantitatively similar to their risk of active disease, which would suggest that much of the impact of smoking takes place during infection. At the same time, one case-control study selected TST-positive controls, thereby comparing patients who were TST positive and had clinical TB to people who were also TST positive but had not progressed to clinical TB [54]; that study also found a strong association between smoking and disease, suggesting that smoking may induce progression or reactivation disease in those infected. We included the outcome TB mortality in this study in order to investigate the association between smoke and TB occurrence rather than the association between smoke and TB treatment outcomes. The risk of death from TB among smokers was found to be somewhat higher than the risk of latent infection or disease, possibly because smoking has been identified as a risk factor for poor TB treatment outcomes among those undergoing therapy [57,72,73].

 There are several potential limitations to this study. First, our findings are based on the results of observational studies; we cannot, therefore, exclude the possibility of confounding by variables that may be associated with each of the exposures. The issue of confounding is particularly a concern in a meta-analysis of observational studies when effect sizes are relatively small, as was the case in the studies considered in this analysis [74]. We therefore performed a stratified analysis to explore the degree to which potential confounders may have influenced the findings. Among possible confounders, alcohol use is a known risk factor for TB and is closely associated with tobacco use in many populations. Those studies that adjusted for alcohol intake in a multivariable model found that the effect of smoking was reduced, but not eliminated. Those studies that controlled for the effect of alcohol were also less heterogeneous as a group than those that did not, a finding which suggests that some of the variability may have resulted from differences in alcohol consumption. Other risk factors that may confound the association between smoking, passive smoking, and IAP and TB include socioeconomic status, gender, and age. Although it is impossible to fully exclude bias introduced by residual confounding, we found that the effects the exposures on TB remained after adjustment for these factors.

More than half of the studies in our review are case-control studies. These used different approaches to the selection of controls, including sampling from hospitals and clinics, from household members, and from the community. Since smoking is associated with a wide range of diseases, the choice of hospital- or clinic-based sampling may lead to over-representation of smokers among the controls, thereby biasing the results toward the null. Similarly, since people dwelling in the same household may share behavioral risk factors, controls chosen from households of smoking TB patients may have been more likely to smoke than would the general population [75]. When we compared the effect estimates for studies stratified on the basis of the control selection strategy, we found that studies that had not used population-based controls tended to report lower effect estimates, consistent with our expectation of a bias toward the null among studies that used hospital- and household-based controls.

Other potential sources of bias include possible misclassification of both exposure and outcome status. The assessment of tobacco smoking relied on self-reported behavior, which may not have been accurate especially among those who consider smoking to be stigmatizing, such as women in some cultural settings. The exposure “current smoking” may also have been subject to reverse causation. Patients are often diagnosed with TB months or more after having first experienced symptoms of the disease, which may cause some patients to quit smoking. This is consistent with the finding of several studies that “former” smoking to be a stronger risk factor for TB than current smoking [34,42,48]. Nonetheless, since “former” smoking also included very distant smoking, both current and former smoking may underestimate the effect of smoking that had occurred just prior to the onset of disease. Similarly, misclassification of passive smoking and IAP may have introduced a bias toward the null in our analysis. The classification of passive smoking among children, for example, relied on parent reports, which may have been influenced by guilt or shame at having exposed the child to an agent suspected of causing disease. Most problematic among exposures was the classification of IAP; this usually relied on the proxy “use of biomass cooking fuel,” which probably only coarsely captured the actual exposure to inhaled smoke. For example, one study that found no association between biomass fuel use and TB noted that houses in the area were well ventilated, and thus actual exposure to inhaled smoke among those using biomass fuels was probably lower.

Misclassification of outcome may have also introduced bias into this analysis. For example, we included a large mortality study conducted in India in which the odds of death among urban male smokers was 4.5 times that of nonsmokers. Since diagnosis of TB in India relies heavily on radiographic findings, TB may be overdetected, especially among patients with pulmonary lesions—such as malignancies—that may be causally linked to smoking [76]. When we repeated our analysis excluding the two Indian mortality studies, the heterogeneity among the remaining studies was reduced. Similarly, when the mortality studies were excluded from the funnel plot, there was much less variability among the studies with the smallest standard errors. Another possible source of outcome misclassification was suggested by Plant and colleagues [32], who noted that the frequency of small induration sizes among TSTs was higher among smokers than nonsmokers, suggesting that smokers may be less capable than nonsmokers of eliciting a vigorous skin test reaction and that latent TB infection in smokers may thus be underdetected when the 10 mm cutoff is used. Despite this possible limitation, we found that the two studies of latent infection that used 5 mm cutoffs for the TST [32,33] reported effects that were not statistically different from those that used 10 mm [30,31,34,35]. Finally, the diagnosis of TB in children is notoriously difficult; if children exposed to passive smoke were more likely to be successfully diagnosed with disease than those who were not, this might have introduced a bias that would explain the strong positive association between passive smoking and TB.

Although our evidence suggests that tobacco smoking is only a moderate risk factor in TB, the implication for global health is critical. Because tobacco smoking has increased in developing countries where TB is prevalent, a considerable portion of global burden of TB may be attributed to tobacco smoking (see Text S1 for an illustrative calculation of population-attributable fraction and attributable deaths in different regions of the world). More importantly, this association implies that smoking cessation might provide benefits for global TB control in addition to those for chronic diseases.

Despite heterogeneity in design, measurement, and quantitative effect estimates among the studies included in this analysis, we found consistent evidence for an increased risk of TB as a result of smoking, with more limited but consistent evidence for passive smoking and IAP as risk factors. These findings suggest that TB detection might benefit from information on exposure to respirable pollutants from sources such as smoking and biomass use, and that TB control might benefit from including interventions aimed at reducing tobacco and IAP exposure, especially among those at high risk for exposure to the infection.

## Supporting Information

Text S1 Population-Attributable Fraction and Attributable Death Due to Tobacco Smoking on TB Mortality in Different Regions of the World (33 KB DOC)

Alternative Language Abstract S1 Translation of the Article into Chinese by Hsien-Ho Lin (64 KB PDF)

## Abbreviations

AM: alveolar macrophage
CI: confidence interval
IAP: indoor air pollution from biomass fuels
OR: odds ratio
TB: tuberculosis
TST: tuberculin skin test

## References

[pmed-0040020-b001] World Health Organization (1997). Tobacco or health: A global status report.

[pmed-0040020-b002] Gajalakshmi V, Peto R, Kanaka TS, Jha P (2003). Smoking and mortality from tuberculosis and other diseases in India: Retrospective study of 43000 adult male deaths and 35000 controls. Lancet.

[pmed-0040020-b003] Maurya V, Vijayan VK, Shah A (2002). Smoking and tuberculosis: An association overlooked. Int J Tuberc Lung Dis.

[pmed-0040020-b004] Davies PD, Yew WW, Ganguly D, Davidow AL, Reichman LB (2006). Smoking and tuberculosis: The epidemiological association and immunopathogenesis. Trans R Soc Trop Med Hyg.

[pmed-0040020-b005] Greenland S (1987). Quantitative methods in the review of epidemiologic literature. Epidemiol Rev.

[pmed-0040020-b006] Higgins JP, Thompson SG, Deeks JJ, Altman DG (2003). Measuring inconsistency in meta-analyses. Br Med J.

[pmed-0040020-b007] Stroup DF, Berlin JA, Morton SC, Olkin I, Williamson GD (2000). Meta-analysis of observational studies in epidemiology: A proposal for reporting. Meta-analysis Of Observational Studies in Epidemiology (MOOSE) group. J Am Med Assoc.

[pmed-0040020-b008] DerSimonian R, Laird N (1986). Meta-analysis in clinical trials. Control Clin Trials.

[pmed-0040020-b009] Begg CB, Mazumdar M (1994). Operating characteristics of a rank correlation test for publication bias. Biometrics.

[pmed-0040020-b010] Egger M, Davey Smith G, Schneider M, Minder C (1997). Bias in meta-analysis detected by a simple, graphical test. Br Med J.

[pmed-0040020-b011] Dong B, Ge N, Liu G (2001). [Social economical status, behaviors and enviroment as the risk factors of tuberculosis in Chengdu China]. Zhonghua Liu Xing Bing Xue Za Zhi.

[pmed-0040020-b012] Mishra VK, Retherford RD, Smith KR (1999). Cooking with biomass fuels increases the risk of tuberculosis. Natl Fam Health Surv Bull.

[pmed-0040020-b013] Malhotra P, Abrol A, Kaur V, Dhar S, Singh A (1996). Prevalence of tuberculosis in Kishtwar Tehsil of Jammu region in Jammu and Kashmir State. J Indian Med Assoc.

[pmed-0040020-b014] Abal AT, Nair PC, Sugathan TN, Pawar S (2003). Influence of smoking on cutaneous delayed-type hypersensitivity reaction by tuberculin skin test. Respir Med.

[pmed-0040020-b015] Soriano I, Soriano R (2003). Indoor air pollution and tuberculosis: A retrospective study. Glow.

[pmed-0040020-b016] Crofton E, Crofton J (1963). Influence of smoking on mortality from various diseases in Scotland and in England and Wales. An analysis by cohorts. Br Med J.

[pmed-0040020-b017] Gutzwiller F, La Vecchia C, Levi F, Negri E, Wietlisbach V (1989). Smoking, prevalence of disease and health service utilization among the Swiss population. Rev Epidemiol Sante Publique.

[pmed-0040020-b018] Hnizdo E, Murray J (1998). Risk of pulmonary tuberculosis relative to silicosis and exposure to silica dust in South African gold miners. Occup Environ Med.

[pmed-0040020-b019] Jentoft HF, Omenaas E, Eide GE, Gulsvik A (2002). Tuberculin reactivity: Prevalence and predictors in BCG-vaccinated young Norwegian adults. Respir Med.

[pmed-0040020-b020] Woo J, Chan HS, Hazlett CB, Ho SC, Chan R (1996). Tuberculosis among elderly Chinese in residential homes: Tuberculin reactivity and estimated prevalence. Gerontology.

[pmed-0040020-b021] Kuemmerer JM, Comstock GW (1967). Sociologic concomitants of tuberculin sensitivity. Am Rev Respir Dis.

[pmed-0040020-b022] Doll R, Peto R, Wheatley K, Gray R, Sutherland I (1994). Mortality in relation to smoking: 40 years' observations on male British doctors. Br Med J.

[pmed-0040020-b023] Miguez-Burbano MJ, Burbano X, Ashkin D, Pitchenik A, Allan R (2003). Impact of tobacco use on the development of opportunistic respiratory infections in HIV seropositive patients on antiretroviral therapy. Addict Biol.

[pmed-0040020-b024] Nisar M, Williams CS, Ashby D, Davies PD (1993). Tuberculin testing in residential homes for the elderly. Thorax.

[pmed-0040020-b025] Al Kubaisy W, Al Dulayme A, Hashim DS (2003). Active tuberculosis among Iraqi schoolchildren with positive skin tests and their household contacts. East Mediterr Health J.

[pmed-0040020-b026] Gupta RK, Gupta A, Jamwal DS, Suri SP (2002). A socio-epidemiological study of tuberculosis in a rural area. Jk Sci.

[pmed-0040020-b027] Shah SA, Mujeeb SA, Mirza A, Nabi KG, Siddiqui Q (2003). Prevalence of pulmonary tuberculosis in Karachi juvenile jail, Pakistan. East Mediterr Health J.

[pmed-0040020-b028] de March-Ayuela P, Rey-Duran R (1997). Passive smoking and risk of pulmonary tuberculosis in children. Int J Tuberc Lung Dis.

[pmed-0040020-b029] Edwards JH (1957). Contribution of cigarette smoking to respiratory disease. Br J Prev Soc Med.

[pmed-0040020-b030] den Boon S, van Lill SW, Borgdorff MW, Verver S, Bateman ED (2005). Association between smoking and tuberculosis infection: A population survey in a high tuberculosis incidence area. Thorax.

[pmed-0040020-b031] Hussain H, Akhtar S, Nanan D (2003). Prevalence of and risk factors associated with Mycobacterium tuberculosis infection in prisoners, North West Frontier Province, Pakistan. Int J Epidemiol.

[pmed-0040020-b032] Plant AJ, Watkins RE, Gushulak B, O'Rourke T, Jones W (2002). Predictors of tuberculin reactivity among prospective Vietnamese migrants: The effect of smoking. Epidemiol Infect.

[pmed-0040020-b033] Solsona J, Cayla JA, Nadal J, Bedia M, Mata C (2001). Screening for tuberculosis upon admission to shelters and free-meal services. Eur J Epidemiol.

[pmed-0040020-b034] McCurdy SA, Arretz DS, Bates RO (1997). Tuberculin reactivity among California Hispanic migrant farm workers. Am J Ind Med.

[pmed-0040020-b035] Anderson RH, Sy FS, Thompson S, Addy C (1997). Cigarette smoking and tuberculin skin test conversion among incarcerated adults. Am J Prev Med.

[pmed-0040020-b036] Gupta BN, Mathur N, Mahendra PN, Srivastava AK, Swaroop V (1997). A study of household environmental risk factors pertaining to respiratory diseases. Energy Environment Monitor.

[pmed-0040020-b037] Yu GP, Hsieh CC, Peng J (1988). Risk factors associated with the prevalence of pulmonary tuberculosis among sanitary workers in Shanghai. Tubercle.

[pmed-0040020-b038] Adelstein AM, Rimington J (1967). Smoking and pulmonary tuberculosis: an analysis based on a study of volunteers for mass miniature radiography. Tubercle.

[pmed-0040020-b039] Shah JR, Warawadekar MS, Deshmukh PA, Phutane PN (1959). Institutional survey of pulmonary tuberculosis with special reference to smoking habits. Indian J Med Sci.

[pmed-0040020-b040] Lienhardt C, Fielding K, Sillah JS, Bah B, Gustafson P (2005). Investigation of the risk factors for tuberculosis: A case-control study in three countries in West Africa. Int J Epidemiol.

[pmed-0040020-b041] Leung CC, Yew WW, Chan CK, Tam CM, Lam CW (2003). Smoking and tuberculosis in Hong Kong. Int J Tuberc Lung Dis.

[pmed-0040020-b042] Shetty N, Shemko M, Vaz M, D'Souza G (2006). An epidemiological evaluation of risk factors for tuberculosis in South India: A matched case control study. Int J Tuberc Lung Dis.

[pmed-0040020-b043] Buskin SE, Gale JL, Weiss NS, Nolan CM (1994). Tuberculosis risk factors in adults in King County, Washington, 1988 through 1990. Am J Public Health.

[pmed-0040020-b044] Lewis JG, Chamberlain DA (1963). Alcohol consumption and smoking habits in male patients with pulmonary tuberculosis. Br J Prev Soc Med.

[pmed-0040020-b045] Brown KE, Campbell AH (1961). Tobacco, alcohol and tuberculosis. Brit J Dis Chest.

[pmed-0040020-b046] Jick SS, Lieberman ES, Rahman MU, Choi HK (2006). Glucocorticoid use, other associated factors, and the risk of tuberculosis. Arthritis Rheum.

[pmed-0040020-b047] Wang GJ, Sleigh A, Zhou G, Jackson S, Liu XL (2005). [Nonbiologic risk factors of pulmonary tuberculosis among adults in Henan: A case-control study]. Zhonghua Liu Xing Bing Xue Za Zhi.

[pmed-0040020-b048] Crampin AC, Glynn JR, Floyd S, Malema SS, Mwinuka VK (2004). Tuberculosis and gender: Exploring the patterns in a case control study in Malawi. Int J Tuberc Lung Dis.

[pmed-0040020-b049] Kolappan C, Gopi PG (2002). Tobacco smoking and pulmonary tuberculosis. Thorax.

[pmed-0040020-b050] Tekkel M, Rahu M, Loit HM, Baburin A (2002). Risk factors for pulmonary tuberculosis in Estonia. Int J Tuberc Lung Dis.

[pmed-0040020-b051] Tocque K, Bellis MA, Beeching NJ, Syed Q, Remmington T (2001). A case-control study of lifestyle risk factors associated with tuberculosis in Liverpool, North-West England. Eur Respir J.

[pmed-0040020-b052] Gupta D, Aggarwal AN, Kumar S, Jindal SK (2001). Smoking increases risk of pulmonary tuberculosis. J Environ Med.

[pmed-0040020-b053] Dong B, Ge N, Zhou Y (2001). [Smoking and alchohol as risk factor of pulmonary tuberculosis in Chengdu: a matched case-control study]. Hua Xi Yi Ke Da Xue Xue Bao.

[pmed-0040020-b054] Alcaide J, Altet MN, Plans P, Parron I, Folguera L (1996). Cigarette smoking as a risk factor for tuberculosis in young adults: A case-control study. Tuber Lung Dis.

[pmed-0040020-b055] Ariyothai N, Podhipak A, Akarasewi P, Tornee S, Smithtikarn S (2004). Cigarette smoking and its relation to pulmonary tuberculosis in adults. Southeast Asian J Trop Med Public Health.

[pmed-0040020-b056] Lowe CR (1956). An association between smoking and respiratory tuberculosis. Br Med J.

[pmed-0040020-b057] Leung CC, Li T, Lam TH, Yew WW, Law WS (2004). Smoking and tuberculosis among the elderly in Hong Kong. Am J Respir Crit Care Med.

[pmed-0040020-b058] Sitas F, Urban M, Bradshaw D, Kielkowski D, Bah S (2004). Tobacco attributable deaths in South Africa. Tob Control.

[pmed-0040020-b059] Lam TH, Ho SY, Hedley AJ, Mak KH, Peto R (2001). Mortality and smoking in Hong Kong: Case-control study of all adult deaths in 1998. Br Med J.

[pmed-0040020-b060] Liu BQ, Peto R, Chen ZM, Boreham J, Wu YP (1998). Emerging tobacco hazards in China: 1. Retrospective proportional mortality study of one million deaths. Br Med J.

[pmed-0040020-b061] Gupta PC, Pednekar MS, Parkin DM, Sankaranarayanan R (2005). Tobacco associated mortality in Mumbai (Bombay) India. Results of the Bombay Cohort Study. Int J Epidemiol.

[pmed-0040020-b062] Tipayamongkholgul M, Podhipak A, Chearskul S, Sunakorn P (2005). Factors associated with the development of tuberculosis in BCG immunized children. Southeast Asian J Trop Med Public Health.

[pmed-0040020-b063] Altet MN, Alcaide J, Plans P, Taberner JL, Salto E (1996). Passive smoking and risk of pulmonary tuberculosis in children immediately following infection. A case-control study. Tuber Lung Dis.

[pmed-0040020-b064] Singh M, Mynak ML, Kumar L, Mathew JL, Jindal SK (2005). Prevalence and risk factors for transmission of infection among children in household contact with adults having pulmonary tuberculosis. Arch Dis Child.

[pmed-0040020-b065] Mishra VK, Retherford RD, Smith KR (1999). Biomass cooking fuels and prevalence of tuberculosis in India. Int J Infect Dis.

[pmed-0040020-b066] Perez-Padilla R, Perez-Guzman C, Baez-Saldana R, Torres-Cruz A (2001). Cooking with biomass stoves and tuberculosis: A case control study. Int J Tuberc Lung Dis.

[pmed-0040020-b067] Houtmeyers E, Gosselink R, Gayan-Ramirez G, Decramer M (1999). Regulation of mucociliary clearance in health and disease. Eur Respir J.

[pmed-0040020-b068] Sopori M (2002). Effects of cigarette smoke on the immune system. Nat Rev Immunol.

[pmed-0040020-b069] Wang H, Yu M, Ochani M, Amella CA, Tanovic M (2003). Nicotinic acetylcholine receptor alpha7 subunit is an essential regulator of inflammation. Nature.

[pmed-0040020-b070] Fick RB, Paul ES, Merrill WW, Reynolds HY, Loke JS (1984). Alterations in the antibacterial properties of rabbit pulmonary macrophages exposed to wood smoke. Am Rev Respir Dis.

[pmed-0040020-b071] Boelaert JR, Gomes MS, Gordeuk VR (2003). Smoking, iron, and tuberculosis. Lancet.

[pmed-0040020-b072] Thomas A, Gopi PG, Santha T, Chandrasekaran V, Subramani R (2005). Predictors of relapse among pulmonary tuberculosis patients treated in a DOTS programme in South India. Int J Tuberc Lung Dis.

[pmed-0040020-b073] Altet-Gomez MN, Alcaide J, Godoy P, Romero MA, Hernandez del Rey I (2005). Clinical and epidemiological aspects of smoking and tuberculosis: A study of 13,038 cases. Int J Tuberc Lung Dis.

[pmed-0040020-b074] Shapiro S (1997). Is meta-analysis a valid approach to the evaluation of small effects in observational studies?. J Clin Epidemiol.

[pmed-0040020-b075] Robins JM, Gail MH, Lubin JH (1986). More on “Biased selection of controls for case-control analyses of cohort studies.”. Biometrics.

[pmed-0040020-b076] Khatri GR, Frieden TR (2000). The status and prospects of tuberculosis control in India. Int J Tuberc Lung Dis.

