# Supplementary material for: Tobacco Smoke, Indoor Air Pollution and Tuberculosis: A Systematic Review and Meta-Analysis
Source: PLoS Med. 2007 Jan 16;4(1):e20. doi: 10.1371/journal.pmed.0040020 (PMC1769410; doi:10.1371/journal.pmed.0040020)
Supplement: Supplementary file 1 [file pmed.0040020.sd002.pdf]

# 吸煙，室內空氣污染與肺結核—系統性回顧與統合分析


## 摘要

背景

主動吸煙、被動吸煙與生質燃料造成的室內空氣污染被認爲是造成結核病潛伏感染，臨床疾病與死亡的危險因子。在世界上結核病的高流行區，煙草與室內空氣污染的危害卻持續存在甚至日益惡化，因此，本研究針對該危害與結核病的相關性，做一系統性回顧與統合分析。

方法/主要發現

本系統性回顧與統合分析納入的研究類型爲觀察性研究，包含主動吸煙、被動吸煙或室內空氣污染與結核病的關連性，並有報告相對危險性與 95%信賴區間的研究，經篩選後而納入，共計有 33 項研究針對主動吸煙，五項研究針對被動吸煙，與五項研究針對室內空氣污染之暴露。具體的證據顯示，無論研究的是結核病潛伏感染，臨床疾病或是死亡，主動吸煙與結核病均有正相關。與非吸煙者比較，主動吸煙者罹患結核病潛伏感染，臨床疾病或是死亡的危險性均呈現增加的趨勢。同樣的，被動吸煙與室內空氣污染也增加臨床上結核病的風險，只是目前文獻上的證據上不如主動吸煙般的強烈。

結論/重要性

一致性的證據顯示主動吸煙增加結核病的風險。而被動吸煙與生物質能所造成的室內空氣污染，似乎也會增加結核病的危險性，只是目前還需要更多的研究來確認這兩項暴露的風險。我們建議：在肺結核的防治策略當中，應當納入戒煙與降低室內空氣污染的項目，尤其是針對容易接觸到結核病的高危險群。 。



**關鍵字: 結核病，主動吸煙，被動吸煙，室內空氣污染，全球健康**
