# Supplementary material for: Tobacco Smoke, Indoor Air Pollution and Tuberculosis: A Systematic Review and Meta-Analysis
Source: PLoS Med. 2007 Jan 16;4(1):e20. doi: 10.1371/journal.pmed.0040020 (PMC1769410; doi:10.1371/journal.pmed.0040020)
Supplement: Supplementary file 2 [file pmed.0040020.sd001.doc]

**Population Attributable Fraction and Attributable Death Due to Tobacco Smoking on TB Mortality in Different Regions of the World**

In order to calculate the population burden of TB mortality attributable to tobacco smoking, we calculated population attributable fraction (PAF) by gender in different regions of the world. The formula of PAF is given by:

$$PAF=\frac{P(RR-1)}{P(RR-1)+1}$$

where P is the gender-and-region-specific smoking prevalence (Jha, Ranson et al. 2002), and RR is the relative risk. To calculate the number of deaths attributable to smoking (attributable death, AD), we multiplied the the estimated PAFs by gender-and-region-specific TB deaths above age 15 (Lopez, Mathers et al. 2006).

The estimates are shown in the following table:

|  | **Male** | | **Female** | |
| --- | --- | --- | --- | --- |
|  | **PAF** | **AD** | **PAF** | **AD** |
| **High-income** | 27.0% | 2791 | 17.4% | 953 |
| **EAP** | 38.3% | 130855 | 4.8% | 8680 |
| **ECA** | 34.6% | 18913 | 13.8% | 1498 |
| **LAC** | 28.1% | 7882 | 18.0% | 2706 |
| **MENA** | 27.5% | 3237 | 6.5% | 578 |
| **SA** | 16.7% | 61559 | 1.0% | 2106 |
| **SSA** | 21.9% | 43501 | 7.4% | 6085 |

Notes:

1. The relative risk of tobacco smoking on TB mortality was set at 2.0 based on the evidence in this meta-analysis.

2. EAP: East Asia and Pacific, ECA: Europe and Central Asia, LAC: Latin America and the Caribbean, MENA; Middle East and North Africa, SA: South Asia, SSA: Sub-Saharan Africa. For all geographical regions, high income countries have been excluded and are shown as a single group.

3. Data source:

a. Gender-and-region-specific smoking prevalence: Jha P, Ranson MK, Nguyen SN, Yach D (2002) Estimates of global and regional smoking prevalence in 1995, by age and sex. Am J Public Health 92: 1002-1006.

b. Gender-and-region-specific TB deaths: Lopez AD, Mathers CD, Ezzati M, Jamison DT, Murray CJL, eds. Global burden of disease and risk factors. New York: World Bank and Oxford University Press; 2006.
